# Systemic Molecularly Targeted Therapies for Neoadjuvant and Salvage Craniopharyngioma: A Contemporary Narrative Review

**DOI:** 10.3390/biomedicines14030499

**Published:** 2026-02-25

**Authors:** Joseph J. Neubecker, Daniel W. Griepp, Jeffrey P. Turnbull, Joshua Caskey, Shivum Desai, Adam Mansour, Rabia Ahmed, Andrew Beggs, Annie T. K. Griepp, Heather Heitkotter, Chad F. Claus, Boyd F. Richards, Prashant S. Kelkar

**Affiliations:** Division of Neurosurgery, Henry Ford Health System Providence Hospital, College of Human Medicine, Michigan State University, Southfield, MI 48075, USA

**Keywords:** papillary craniopharyngioma, adamantinomatous craniopharyngioma, *BRAF V600E*, MEK inhibitor, IL-6/IL-6 receptor blockade, bevacizumab, targeted therapy, salvage therapy

## Abstract

Craniopharyngiomas are rare, histologically benign but locally aggressive intracranial tumors that are associated with substantial visual, endocrine, and hypothalamic morbidity. Advances in molecular characterization have enabled the use of systemic molecularly targeted therapies, particularly in the recurrent or refractory setting, with the goal of limiting further surgical or radiation-related injury to the hypothalamic–pituitary axis. Papillary craniopharyngioma (PCP), defined by near-universal *BRAF V600E* mutations, exhibits profound and rapid responses to combined BRAF and MEK inhibition, with objective response rates exceeding 90% in prospective studies. These responses can facilitate less extensive surgery, enable de-escalation of radiotherapy, or allow deferral of local treatment. In contrast, adamantinomatous craniopharyngioma (ACP), characterized by CTNNB1 mutations and a cystic phenotype with a prominent inflammatory microenvironment, lacks a single actionable oncogenic driver. Early clinical experience suggests that Interleukin-6/Interleukin-6 receptor (IL-6/IL-6R) blockade, alone or in combination with bevacizumab, may stabilize or reduce cystic components in selected patients, although evidence remains limited to small case series. Other systemic approaches for ACP, including MAPK pathway inhibition and immune-directed strategies, are still under investigation. Across subtypes, adverse events have generally been class-expected and manageable, but data on long-term endocrine, hypothalamic, and neurocognitive outcomes are sparse. This review synthesizes current evidence for neoadjuvant, adjuvant, and palliative craniopharyngioma systemic targeted therapies and highlights the ongoing clinical considerations of this therapy.

## 1. Introduction

Craniopharyngiomas are rare epithelial tumors arising from remnants of Rathke’s pouch in the sellar and suprasellar region, accounting for 1–5% of intracranial neoplasms (with most series reporting 2–5%) [1,2,3]. Although histologically benign, their proximity to and frequent involvement of the optic apparatus, hypothalamus, and pituitary gland confers a high risk of morbidity related to both tumor progression and treatment [3]. Two biologically and clinically distinct tumor types are recognized: papillary craniopharyngioma (PCP), which predominates in adults and harbors *BRAF V600E* mutations in approximately 95% of cases, and adamantinomatous craniopharyngioma (ACP), which more commonly affects children and young adults and is characterized by CTNNB1 mutations in 69–100% of cases, affecting the Wnt/beta-catenin signaling pathway and with a radiographically cystic growth pattern [3,4,5,6,7,8,9,10].

For decades, management for both subtypes has centered on gross total resection when safely achievable, though this approach has increasingly shifted toward limited, hypothalamus-sparing resection followed by radiotherapy in cases with hypothalamic involvement [1,3,11]. Nevertheless, survivors of childhood-onset craniopharyngioma experience high rates of severe obesity, metabolic disease, and impaired quality of life consequent of both tumor involvement and treatment-related damage to posterior hypothalamic structures [11,12,13,14,15].

These limitations have driven interest in systemic therapies capable of controlling tumor growth while minimizing further damage to critical neuroendocrine structures [16,17,18]. Moreover, recurrence still occurs, despite gross total resection or radiotherapy. Given the substantial morbidity of revision craniopharyngioma surgery and recurrent radiation therapy, systemic targeted therapies for recurrence or progression are increasingly utilized as hypothalamus-sparing salvage options [19,20]. *BRAF/MEK* inhibitor therapy has substantially altered the therapeutic landscape for PCP, while inhibition of the interleukin-6 pathway has shown promise in the management of cystic components of ACP. This review synthesizes current evidence for neoadjuvant, adjuvant, and palliative craniopharyngioma systemic targeted therapies and highlights the ongoing clinical considerations of these therapies.

## 2. Background

### 2.1. Population and Epidemiology

ACP demonstrates a bimodal age distribution with peaks at 5–15 years and 45–60 years, making it the predominant subtype in the pediatric population [3,4]. In contrast, PCP is most frequently observed in adults, primarily occurring in the fifth and sixth decades of life [3]. While PCP was historically considered exclusively an adult disease, sporadic pediatric cases with confirmed *BRAF V600E* mutations have been reported since 2018, though these remain rare (approximately 3% of all PCP cases) [21].

### 2.2. Molecular and Genetic Profiles

The two tumor types of craniopharyngiomas harbor mutually exclusive genetic alterations that define their molecular pathogenesis [7,22]. ACP is driven by somatic mutations in *CTNNB1* (encoding β-catenin) with mutations predominantly occurring in exon 3 [7,22]. These mutations result in pathologic stabilization of β-catenin and constitutive activation of the Wnt/beta-catenin signaling pathway [23,24]. Some evidence suggests mutations at *Thr41* may correlate with worse event-free survival [8,22]. Importantly, next-generation sequencing studies have not identified other recurrent mutations in ACP beyond *CTNNB1*, indicating this is the crucial pathogenetic driver [22].

PCP is characterized by *BRAF V600E* mutations in 94–95% of cases, which activate the RAS/RAF/MEK/ERK (MAPK) pathway [7,22]. This mutation is clonal and represents the primary oncogenic driver, with no other recurrent genomic alterations identified [25]. The presence of *BRAF V600E* has critical therapeutic implications, as these tumors demonstrate remarkable responsiveness to combined BRAF and MEK inhibitor therapy [7,8].

Recent multi-omics analyses have suggested additional molecular heterogeneity within ACP, identifying three possible molecular subgroups: Wnt (characterized by high Wnt/beta-catenin pathway activity), ImA (with activated inflammatory pathways and MEK/MAPK signaling), and ImB (showing elevated Interleukin-6 (IL-6) expression and epithelial–mesenchymal transition markers) [26,27]. These subgroups correlate with different imaging features, histological characteristics, and clinical outcomes, with the Wnt subgroup demonstrating longer event-free survival compared to ImB [17,26]. Genomic analysis has shown that most PCP and a subset of ACP have stable genomes, while other ACP cases demonstrate focal chromosomal gains or losses in specific regions such as Xq28 [22,23,28,29,30].

## 3. Therapies

### 3.1. Papillary Craniopharyngioma Treatment

In *BRAF V600E*–mutant PCP, dual BRAF/MEK inhibition has shown high objective response rates in early prospective studies (Table 1). In the landmark Alliance A071601 phase II trial of neoadjuvant therapy with vemurafenib (a inhibitor of mutated *BRAF V600E* kinase,) plus cobimetinib (a MEK1 and MEK2 inhibitor), 15/16 patients (94%; 95% CI, 70–100) achieved an objective partial or better response at 4 months by centrally reviewed volumetric MRI, with a median tumor-volume reduction of 91% (range, 68–99) [7]. In the French multicenter cohort of dabrafenib (competitive inhibitor of BRAF kinase, specifically V600E, V600K, and V600D BRAF proteins) plus trametinib (another MEK1 and MEK2 inhibitor) (*n* = 16; neoadjuvant/adjuvant/palliative), 94% demonstrated partial or better response. Follow-up responses were categorized as subtotal response (12/16), partial response (3/16), and stable disease (1/16); mean volume reduction was 88.9% (neoadjuvant), 73.3% (adjuvant), and 91.8% (palliative) [31]. A recent systematic review of 21 studies, including 54 patients, similarly reported median tumor volume reduction of 89% overall (neoadjuvant 90%, adjuvant 85%, and palliative 88%), and volumetric response rates as high as 94% (Alliance) and 93% (French) [32].

Additionally, disease control appears closely linked to therapy compliance. Follow-up from Alliance A071601 indicated that progression-free survival was 87% at 12 months and 58% at 24 months [7]. Importantly, no patient progressed while receiving per-protocol vemurafenib–cobimetinib and among patients who discontinued protocol therapy, 6/7 had no evidence of progression at a median 23-month follow-up [7]. Rapid recurrence within 3 months is typical following treatment discontinuation; however, tumors remain responsive to therapy reinitiation without evidence of acquired resistance even after multiple treatment courses [33]. Case reports further describe and confirm tumor regrowth after interruption and renewed response upon rechallenge, suggesting persistent oncogenic dependence on MAPK signaling [7].

It should also be noted that tumor morphology influences response, with mixed-type tumors demonstrating significantly faster and more robust responses than purely solid or cystic lesions [34,35]. This may reflect differential drug penetration and the observation that enhancing components show greater volume reduction (96%) than cystic components (82%) [2].

**Table 1:** Summary of some recent clinical studies of systemic targeted therapy.

Study	Description	Targeted Therapy	Key Outcomes
Gunasekara et al., 2025 [36]	*n* = 1 (pediatric, multiply progressive ACP with brittle CDI)	Trametinib	Acute severe hyponatremia and reduced desmopressin in adolescents with progressive ACP and brittle central DI. Trametinib was discontinued after 3 months due to treatment failure and ongoing side effects.
De Alcubierre et al., 2024 [31]	*n* = 16 (PCP, mixed setting) Retrospective cohort, multicenter (France)	Dabrafenib + trametinib	Mean TVR: 88.9% (NEO), 73.3% (ADJ), 91.8% (PAL). Headaches resolved in 5/5; vision improved in 6/9.
de Vos-Kerkhof et al., 2023 [37]	*n* = 1 (5th cystic progression of ACP)	Tocilizumab IV 800 mg q2 weeks	Treatment showed good response at ~9 months with clinical stability maintained off therapy (reported follow-up approaching 3 years).
Brastianos et al., 2023 [7]	*n* = 16 (PCP, newly diagnosed) Phase II trial, multicenter (Alliance A071601)	Vemurafenib + cobimetinib	In total, 15/16 (94%) achieved partial response or better by centrally reviewed volumetric MRI; median TVR tumor-volume reduction 91% (range 68–99%) after 4 cycles.
Calvanese et al., 2022 [38]	*n* = 2 adult *BRAFV600E*-mutant PCP (1 recurrent post–near-total resection, 1 newly diagnosed biopsy-only)	Dabrafenib + trametinib	Treatment as adjuvant (case 1) and neoadjuvant (case 2) therapy; a 90% TVR after 5 mos. Both patients underwent fractionated radiotherapy to the small residual tumor with no complications or side effects.
Chik et al., 2021 [39]	*n* = 1 (childhood-onset, recurrent PCP)	Vemurafenib	Treatment for 40 months after multiple surgeries with tumor reduction at 6 wks. Dose reduction due to elevated liver enzymes led to regrowth with recurrence in 7 weeks after stopping therapy. Resumed vemurafenib prior to RT led to tumor shrinkage at 16 days allowing RT completion and tumor control.
Patel et al., 2021 [40]	*n* = 1 (adult, recurrent ACP)	Binimetinib	Brain MRI at 10 months post drug initiation showed significant tumor size reduction. MRI stable at 1 year follow up. Dose-dependent side effects.
Grob et al., 2019 [41]	*n* = 2 (pediatric, recurrent cyst-dominant ACP)	Tocilizumab 12 mg/kg IV every 2 weeks (1 received bevacizumab)	Treatment resulted in reduction in cyst burden. One developed grade 3 neutropenia (dose delays); otherwise, treatment was tolerated.

Tumor-volume reduction: TVR, radiation therapy: RT, diabetes insipidus, DI, neoadjuvant: NEO, adjuvant: ADJ, palliative: PAL.

Studies have also identified that symptom improvement tends to correlate with radiographic shrinkage. In the French cohort, targeted therapy resolved headaches in 5/5 patients and improved visual impairment in 6/9, with endocrine recovery reported in 2/14 [31]. Across published reports, many patients begin targeted therapy with pre-existing pituitary hormone dysfunction following prior surgery and/or radiotherapy, and recovery of established deficits appears uncommon despite marked tumor regression [31,32,42]. Thus, a major practical value of neoadjuvant BRAF/MEK therapy is facilitation of less extensive local therapy; multiple series describe profound volumetric shrinkage that enables limited resection and/or de-escalation of radiotherapy planning (including smaller target volumes), with the goal of minimizing additional hypothalamic injury [7,31,38,42,43].

In neoadjuvant paradigms, tumor-volume reductions of ~85–90% within several months (~3–6 months) have enabled less aggressive surgical approaches and, in selected cases, deferral of surgery [31,38,43]. Conceptually, “medical debulking” aligns with hypothalamus-preservation principles by reducing the need for extensive dissection in anatomically constrained tumors. However, whether this strategy translates into improved long-term hypothalamic outcomes (e.g., obesity risk) remains insufficiently reported and should be evaluated prospectively [11,38,44,45]. Furthermore, adverse events warrant careful monitoring. Across recent series, treatment discontinuation due to toxicity occurred in 14.5–18.8% of patients, with common adverse events including rash, pyrexia, diarrhea, elevated liver enzymes, and occasional cardiotoxicity [31,34,42].

Notably, the 2025 NCCN Pediatric CNS Cancer Guidelines now list dabrafenib/trametinib and vemurafenib as preferred targeted therapies for *BRAF V600E*-mutated tumors in both adjuvant and recurrent/progressive settings [46]. This highlights both the efficacy and widespread adoption of this treatment. Despite these promising results, critical questions remain regarding the optimal duration of targeted therapy, the ideal sequencing with surgery and radiotherapy, and whether all patients require consolidative local treatment following medical debulking [32,47]. The Alliance A071601 trial originally proposed four cycles of therapy followed by definitive treatment, yet many patients continued beyond this timeframe with acceptable toxicity, and some avoided radiation entirely without progression, highlighting the need for prospective studies to establish standardized protocols [48]. Lack of long-term follow-up in this patient population limits conclusions regarding the long-term efficacy of these treatments.

### 3.2. Adamantinomatous Craniopharyngioma Treatment

Targeted treatment for ACP remains primarily investigational and, unlike PCP, does not have a single routinely actionable oncogenic driver analogous to *BRAF V600E*. Most systemic strategies are supported by small clinical reports and early translational data rather than prospective efficacy trials [1,3,6,49]. Prior studies sampling ACP cystic fluid have demonstrated high levels of IL-6, up to 50,000 times that in cerebrospinal fluid [50]. As a driver of pro-inflammatory signaling pathways, IL-6 is associated with cyst formation, driving tumor progression, and local tissue invasion [50,51]. The robust inflammatory response driven by IL-6 is a suspected contributor to ACP’s tendency to adhere to adjacent structures such as the optic chiasm and hypothalamus. Targeting IL-6 with IL-6/IL-6 receptor blockade (tocilizumab ± bevacizumab) has generated the most reproducible published systemic signal to date in ACP, largely through cyst control rather than durable eradication of solid tumor [18,37,41,52].

Grob et al. described two pediatric patients with recurrent cystic disease who were treated with tocilizumab (± bevacizumab) and experienced meaningful reductions in cyst burden [41]. Another report described prolonged stabilization of cystic progression with biweekly intravenous tocilizumab in a pediatric patient [37]. Webb et al. published a recent case demonstrating cyst control with tocilizumab + bevacizumab in recurrent ACP [53]. 

Overall, IL-6/IL-6R blockade appears to be a promising non-surgical strategy to manage cyst accumulation, and although solid nodules commonly persist, this may potentially defer higher-risk local interventions in well-selected patients [37,41,52,53]. The CONNECT 1905 trial (NCT05233397) is a phase II clinical trial currently enrolling patients aged 1 to 25 years to evaluate the effects of tocilizumab on progressive or recurrent ACP [54].

Safety data remain limited; reported adverse events were class-expected, including grade 3 neutropenia, underscoring the need for systematic prospective monitoring [37,41,53].

### 3.3. Experimental Therapy with MAPK/ERK–MEK Pathway Inhibition

In contrast to PCP, ACP treatment with MAPK/ERK targeting has not yet demonstrated consistent clinical benefit. Molecular profiling and compartment-level studies show MAPK/ERK pathway activation in ACP, particularly in recurrent disease, providing biologic rationale for MEK inhibition even without *BRAF V600E* mutations [2,35]. In explant models, MEK inhibition (e.g., trametinib) reduced proliferation and increased apoptosis, supporting translational interest [55]. The CONNECT 2108 trial (NCT05286788) is an active phase 2 clinical study evaluating binimetinib, an oral MEK1/2 inhibitor, in pediatric ACP [54]. Another trial is the CONNECT 2103 trial (NCT05465174), currently enrolling pediatric patients with recurrent ACP, stratified by prior radiotherapy status, to receive binimetinib with radiological response as the primary endpoint [10]. While clinical trials evaluating MEK inhibitors are underway, clinical experience remains very limited, with potential safety concerns in patients with fragile hypothalamic-pituitary physiology [56].

Notably, a pediatric patient with ACP and central diabetes insipidus developed severe hyponatremia with reduced desmopressin requirements shortly after initiating trametinib; rapid rebound effects upon trametinib cessation prevented evaluation of tumor response [36]. Similar hyponatremia has been reported in pediatric low-grade glioma patients with diabetes insipidus receiving trametinib, potentially related to MEK inhibitor effects on aquaporin channel physiology [57]. A case report describing use of binimetinib for recurrent ACP with Wnt mutation demonstrated good radiographic and clinical response; however, requiring frequent dose adjustments due to side effects of uruncles/papulopustular rash, hyponatremia, poor wound healing, and weight gain [40]. Given sparse efficacy data and potential toxicity with variable side effect profile, MEK inhibitor monotherapy should be considered experimental and preferentially pursued within clinical trials [28,36,54].

### 3.4. Other Therapies 

Investigation into the immune microenvironment of craniopharyngioma has also revealed potential immunotherapeutic targets. Immunohistochemical studies demonstrate frequent expression of immune-related targets in craniopharyngioma, including programed cell death (PD)-L1 and high B7-H3 expression, suggesting an immunologically active and potentially targetable microenvironment [58]. B7-H3 overexpression has been independently reported, and organoid models support the tractability of B7-H3-directed strategies, with therapeutic effects varying by modality, thereby providing a rationale for further development. [59,60]. The Pediatric Neuro-oncology Consortium trial (PNOC029) is evaluating tovorafenib (pan-RAF inhibitor) monotherapy and in combination with nivolumab (PD-1 receptor inhibitor) in patients up to 39 years of age with newly diagnosed or recurrent ACP, using a composite primary endpoint of progression-free survival and quality of life at 12 months with extended follow-up continuing for 3 years after treatment completion [10,61].

Nevertheless, ACP-specific prospective clinical efficacy data for checkpoint inhibitors remain limited, and these agents remain under investigation [52,54]. When considering treatment, clinicians must weigh immune-related endocrinopathies (including hypophysitis and other endocrine toxicities) that could compound baseline hypothalamic–pituitary morbidity in this population [62,63].

## 4. Clinical Implications

Our review highlights BRAF-/MEK-targeted therapy as an increasingly utilized in both neoadjuvant and salvage treatment for PCP, whereas the role of targeted therapies in ACP remains under investigation. In clinical practice, adult patients with BRAF-mutant PCP who present with recurrent or unresectable disease should be considered for treatment with combined BRAF/MEK inhibition [47]. In selected cases, neoadjuvant administration can result in substantial tumor reduction, facilitating surgical resection and, in some instances, obviating the need for adjuvant radiotherapy [7,64].

Conversely, for patients with ACP, especially those with symptomatic cystic recurrences, use of tocilizumab may be considered [37,41]. In this context, the therapeutic objective is disease stabilization and symptomatic improvement rather than complete tumor eradication. Given the absence of established systemic therapies targeting the solid component of ACP, enrollment in clinical trials should be pursued whenever feasible [54]. As mentioned, multiple clinical trials are currently investigating targeted therapies for both craniopharyngioma tumor types. For ACP, active clinical trials are mainly exploring MEK inhibition, PD-1 blockade, Wnt pathway targeting, and IL-6 inhibition [54]. For PCP, ongoing studies are focused on investigating *BRAF V600E* and MEK inhibition as single-agent or combination strategies [54]. Other targets have been reported and represent an ongoing area of study. Notably, most current trials in ACP are limited to children and young adults.

Across both craniopharyngioma tumor types, systematic and longitudinal assessment of visual function, endocrine status, hypothalamic regulation of appetite and thirst, cognitive performance, and quality of life is essential during and after therapy [19,65]. As targeted treatments transition from isolated case reports to broader clinical application, close collaboration between treating physicians, endocrinologists, and neuropsychologists is warranted to ensure comprehensive outcome documentation. Clinicians should maintain vigilant surveillance for established treatment-related toxicities—particularly dermatologic and febrile reactions—and manage these proactively, as therapy can often be safely continued with appropriate supportive measures and dose adjustments [18]. Future comparative trials are essential to define treatment duration, determine which patients may safely defer or avoid surgery and radiotherapy after targeted therapy, and optimize integration of these modalities to maximize tumor control while minimizing long-term morbidity.

## 5. Conclusions

BRAF/MEK inhibitor therapy has substantially altered the therapeutic landscape for PCP, while inhibition of the interleukin-6 pathway has shown promise in the management of cystic components of ACP. However, the extent to which these targeted strategies translate into sustained improvements in long-term endocrine and neurocognitive outcomes remains uncertain. These considerations underscore the need for prospective studies incorporating extended follow-up and patient-centered outcome measures.

## Data Availability

No new data were created or analyzed in this study. Data sharing is not applicable to this article.

## Abbreviations

The following abbreviations are used in this manuscript:

PCP	Papillary craniopharyngioma
ACP	Adamantinomatous craniopharyngioma
